# Essential Oil Composition and Bioactivity of Two Juniper Species from Bulgaria and Slovakia

**DOI:** 10.3390/molecules26123659

**Published:** 2021-06-15

**Authors:** Valtcho D. Zheljazkov, Charles L. Cantrell, Ivanka Semerdjieva, Tzenka Radoukova, Albena Stoyanova, Vasilina Maneva, Miroslava Kačániová, Tess Astatkie, Daniela Borisova, Ivayla Dincheva, Ivan Salamon

**Affiliations:** 1 Crop and Soil Science Department, Oregon State University, Corvallis, OR 97331, USA; 2 Natural Products Utilization Research Unit, United States Department of Agriculture–Agricultural Research Service (USDA-ARS), University, MS 38677, USA; charles.cantrell@usda.gov; 3 Department of Botany and Agrometeorology, Agricultural University, 4000 Plovdiv, Bulgaria; v_semerdjieva@abv.bg; 4 Department of Botany and Methods of Biology Teaching, Faculty of Biology, University of Plovdiv Paisii Hilendarski, 4000 Plovdiv, Bulgaria; kiprei@abv.bg; 5 Department of Tobacco, Sugar, Vegetable and Essential Oils, Technological Faculty, University of Food Technologies, 4002 Plovdiv, Bulgaria; aastst@abv.bg; 6 Plant Protection and Technology Department, Institute of Agriculture, Karnobat, Agricultural Academy, 8400 Karnobat, Bulgaria; maneva_ento@abv.bg; 7 Department of Fruit Sciences, Viticulture and Enology, Faculty of Horticulture and Landscape Engineering, Slovak University of Agriculture, 949 76 Nitra, Slovakia; kacaniova.miroslava@gmail.com; 8 Department of Bioenergetics and Food Analysis, Institute of Food Technology and Nutrition, University of Rzeszow, 35-601 Rzeszow, Poland; 9 Department of Engineering, Faculty of Agriculture, Dalhousie University, Truro, NS B2N 5E3, Canada; astatkie@dal.ca; 10 Administration of Vrachanski Balkan Nature Park, Executive Forest Agency, Ministry of Agriculture, Food and Forestry, 3000 Vratsa, Bulgaria; didieco@gmail.com; 11 Plant Genetic Research Group, AgroBioInstitute, Agricultural Academy, 1164 Sofia, Bulgaria; ivadincheva@yahoo.com; 12 Department of Ecology, Faculty of Humanities and Natural Sciences, University of Presov, 080 01 Presov, Slovakia; ivan.salamon@unipo.sk

**Keywords:** protected plants, podophyllotoxin, sabinene, α-pinene, α-cedrol, α-limonene, terpinen-4-ol, elemol, safrol, eugenol, anti-cancer, antimicrobial

## Abstract

*Juniperus excelsa* M. Bieb and *J. sabina* L. contain essential oil (EO), while *J. sabina* also contains podophyllotoxin, which is used as a precursor for anti-cancer drugs. Two studies were conducted. The first assessed the variability in the EO profile and podophyllotoxin concentration of the two junipers, depending on the location and tree gender. The main EO constituents of *J. excelsa* were α-cedrol, α-limonene and α-pinene, while the constituents in *J. sabina* were sabinene, terpinen-4-ol, myrtenyl acetate and α-cadinol. The podophyllotoxin yield of 18 *J. sabina* accessions was 0.07–0.32% (*w/w*), but this was not found in any of the *J. excelsa* accessions. The second study assessed the effect of hydrodistillation (Clevenger apparatus) and steam distillation (in a semi-commercial apparatus) on the EO profile and bioactivity. The extraction type did not significantly alter the EO composition. The EO profiles of the two junipers and their accessions were different and may be of interest to the industry utilizing juniper leaf EO. Breeding and selection programs could be developed with the two junipers (protected species) in order to identify chemotypes with (1) a high EO content and desirable composition, and (2) a high concentration of podophyllotoxin in *J. sabina*. Such chemotypes could be established as agricultural crops for the commercial production of podophyllotoxin and EO.

## 1. Introduction

Juniper (*Juniperus* L.) species are some of the most widely distributed plants on the planet [1,2]. Most junipers are characterized by high ecological plasticity and inhabit diverse areas at various latitudes and altitudes [1]. Although most junipers are slow-growing trees and bushes, juniper wood is durable and valued for its color, aroma and antimicrobial properties. Furthermore, junipers are important for a number of wildlife species, especially in arid regions; they are also very widely used as ornamentals and as a source for natural plant products [3]. Most junipers contain essential oil (EO) with a specific aroma, while some also contain podophyllotoxin [1,4], among other non-volatile compounds. The EO of some juniper species extracted from leaves, wood, or berries (galbuli) is used as a major aromatic agent in numerous consumer products [5,6].

Podophyllotoxin is used in the development of the commercially available anti-cancer drugs etoposide and teniposide, which are used against neuroblastoma, testicular cancer, lung cancer, hepatoma and others [7,8,9], and are also used for the treatment of psoriasis and malaria [10,11]. Currently, podophyllotoxin is commercially isolated from the Himalayan mayapple (*Podophyllum hexandrum* Royle), an endangered species. Some junipers and other species have shown promise as alternative feedstock for podophyllotoxin [4,12,13,14,15,16,17,18,19]. The juniper species in Eastern Europe have not been fully explored as sources for podophyllotoxin and for EOs with specific aromas that would be of interest for the industry.

Although *Juniperus sabina* and *J. excelsa* have limited distribution amongst the Bulgarian and Slovakian flora, they are of interest to various industries and folk medicine. Due to their specific phytochemical composition and pharmacological application, the EO have been used for cosmetic and medical purposes for a long time [20,21,22,23,24,25,26,27,28,29,30,31]. The literature data on the *J. excelsa* and *J. sabina* EO is summarized in Table 1. The hypothesis of this study was that the EO composition and bioactivity of *J. sabina* and *J. excelsa* will depend on its location, sex and species, and will be altered by the distillation type (hydrodistillation (HD) and steam distillation (SD)). A secondary hypothesis was that all of the populations of *J. sabina* contain various amounts of podophyllotoxin, and accessions with relatively high concentrations of podophyllotoxin can be identified.

## 2. Results

Two separate studies were conducted and described in this manuscript: (1) The objective of the first study was to assess the variability in the EO profile and podophyllotoxin concentration of *Juniperus sabina* and *J. excelsa,* depending on the location and sex of the tree. (2) The objective of the second experiment was to assess the effect of the EO extraction (hydrodistillation using a Clevenger-type apparatus vs. steam distillation in a semi-commercial extractor) on the EO profile and bioactivity of *J. sabina* and *J. excelsa*.

### 2.1. Juniperus excelsa and J. sabina Essential Oil (EO) Yield in Different Locations in Bulgaria (the First Experiment)

Overall, the EO yield of the dried leaves of *J. excelsa* (1.16%) was lower than that of *J. sabina* (1.98%) (Table 2). However, the EO yield of both junipers varied among different accessions, from 0.60% to 1.87% in *J. excelsa* and from 1.38% to 2.73% in *J. sabina,* suggesting significant variability occurring within a population (Table 2). These results demonstrated that high- yielding EO accessions of the two junipers could be identified and selected from the natural populations for the potential commercial production of EO.

### 2.2. Juniperus excelsa and J. sabina Essential Oil (EO) Composition in Different Locations in Bulgaria (the First Experiment)

#### 2.2.1. *Juniperus excelsa* EO Composition

Twenty nine (29) constituents were identified in *J. excelsa* EO, and the range of each of these constituents is given in Supplementary Table S1. Please note that while most junipers are dioecious, *J. excelsa* is monoecious; however, some trees do form galbuli and other trees do not. This may be confusing, as in most other junipers that are dioecious, the female trees produce galbuli while the male trees do not. The EO yield of *J. excelsa* collected at three different locations depending on the presence of galbuli varied from 0.69 to 1.87% in the dried material, with significant differences among the locations and depending on the presence of galbuli (Table 2). The three major EO constituents of *J. excelsa* EO were α-cedrol (29.1–32.3%), α-limonene (24.1–26.4%) and α-pinene (19.7–22.5%) (Supplemtary Table S1).

Analysis of variance (ANOVA) was completed to determine the effect of Location–Sex of the 10 main constituents, namely: α-pinene, β-pinene, α-limonene, bornyl acetate, germacrene D, γ-cadinene, germacrene B, caryophyllene oxide, α-cedrol and 1-epi-cubenol (Table 3). The concentrations of α-pinene and α-limonene were the highest in the EO of plants without galbuli from Izgoryaloto Gyune, Rhodope Mountains, and α-limonene was not different from the EO of the plants with galbuli from Kresna Gorge (Table 3). The EO of the plants with galbuli from Kresna Gorge also had the highest concentrations of bornyl acetate, germacrene D, γ-cadinene, germacrene B, caryophyllene oxide and 1-epi-cubenol, whereas the EO of plants from Izgoryaloto Gyune in the Rhodope Mountains with galbuli contained the highest concentration of α-cedrol (Table 3).

Overall, the two major classes of the *J. excelsa* EO constituents were monoterpenes and sesquiterpenes. The amount of monoterpenes was around 49.9–53.8% (Supplementary Table S1). Monoterpene hydrocarbons were the major part of the total monoterpenes, and oxygenated monoterpenes were present in relatively low concentrations of around 1%, with the exception of one of the samples, in which they reached 7.3%. The total sesquiterpenes were 44.4–48.2%. The total sesquiterpenes included tricyclic oxygenated sesquiterpenes as a major group, with monocyclic sesquiterpene hydrocarbons, bicyclic sesquiterpene hydrocarbons and oxygenatedbicyclic sesquiterpenes being in lower concentrations (Supplementary Table S1).

#### 2.2.2. *Juniperus sabina* Essential Oil (EO) Composition Collections in Bulgaria and Slovakia

Forty six (46) EO constituents were identified in the *J. sabina* samples collected in Bulgaria and Slovakia (Supplementary Table S2). Overall, the major EO constituents of *J. sabina* were sabinene (16.68–30.98%), terpinen-4-ol (9.25–13.63%), myrtenyl acetate (1.32–23.02%), elemol (8.45–13.70%) and α-cadinol (3.47–3.77%). Other EO constituents which were above 1% of the total oil included α-thujene, α-pinene, p-cymene, limonene and δ-cadinene. The EO profile of *J. sabina* from Slovakia was not very different; it was generally within the observed profile of the *J. sabina* EO accessions from Bulgaria (Supplementary Table S2). 

The effect of Location–Sex on the essential oil content was not significant, but it was significant on all 19 constituents. The ANOVA assumptions were met by all of the response variables, and a multiple means comparison for the 19 constituents was completed using Fisher’s LSD at the 5% level of significance (Table 4). The EO of F plants of *J. sabina* from Beli Iskar at the Rila Mountains had the highest concentrations of α-thujene, α-pinene, β-myrcene, p-cymene, limonene, cis-sabinene hydrate, β-linalool, β-citronellol, (S)-(-)-citronellic acid methy, myrtenyl acetate, germacrene D, δ-cadinene, α-cadinene, elemol and α-cadinol, whereas the EO of M plants from the same location had the highest concentrations of terpinen-4-ol, methyl eugenol and sclarene, and the EO of M plants from Krushovska bara had the highest concentrations of sabinene (Table 4).

The two major chemical classes of the EO constituents of *J. sabina* were monoterpenes and sesquiterpenes (Supplementary Table S2). The monoterpenes were the highest in the EO from Zvolen, Slovakia, at 73.2%, while the total monoterpenes collected in Bulgaria were 61.3–70.6%. Of the total monoterpenes, the monoterpene hydrocarbons were the largest group (26.9–41.5%), followed by the oxygenated monoterpenes (15.6–36.5%). The total sesquiterpenes comprised 19.97–25.96% of the total oil, with the major ones being the monocyclic sesquiterpene hydrocarbons (10.5–15.9%), oxygenated bicyclic sesquiterpenes (5.4–5.8%) and bicyclic sesquiterpene hydrocarbons (3.5–3.8%) (Supplementary Table S2). The phenylpropanoid compound (methyl eugenol) in the EO from the male plants collected in Beli Iskar Bulgaria was 13.5%, while its concentration in the EO of other *J. sabina* samples was negligible, below 1% (Supplementary Table S2).

### 2.3. Podophyllotoxin Yield (the First Experiment)

The podophyllotoxin yield in the 18 accessions in this study ranged from 0.065% in accession #77 to 0.320% in accession #65 (Table 5).

### 2.4. Second Experiment on Two Different EO Extraction Methods for J. excelsa and J. sabina

#### 2.4.1. *Juniperus excelsa*—Two Different Extraction Methods (the Second Experiment)

Most of the published research on the EO composition of *J. sabina* and *J. excelsa* used the hydrodistillation method using a Clevenger apparatus (Table 1). Indeed, this method has been a standard laboratory practice for a long time because of its simplicity and efficient EO extraction. However, the industry is extracting juniper EO using steam distillation due to the much wider availability of commercial steam distillation extraction facilities relative to hydrodistillation ones. Although there have been comparative studies between the two extraction methods for other aromatic plants [23,24], these have not been compared for the two juniper species subject to this study. Therefore, in order to test the hypothesis that the commonly used laboratory method (hydrodistillation method using a Clevenger apparatus) would provide a differential EO composition compared with the steam distillation used by the industry, we extracted subsamples from the same larger samples using both methods, as described in the Materials and Methods section.

Overall, the two EO extraction methods provided a very similar EO profile for *J. excelsa* (Table 6). Of the 36 EO constituents identified in *J. excelsa* in this study, α-cedrol (24.06–27.00%) and α-limonene (23.23–27.50%) were the main ones, followed by α-pinene (18.90–22.30%). The other EO constituents with concentrations above 1% of the total oil included α-cedrene, β-caryophyllene, γ-elemene, germacrene D, δ-cadinene and 1-epi-cubenol (Table 6).

The major classes of the *J. excelsa* EO compounds from the second experiment were monoterpenes and sesquiterpenes, both at around 50% of the total oil (Table 6). The monoterpene hydrocarbons (about 49%) were the major portion of the total monoterpenes, with the oxygenated monoterpenes and the bicyclic oxygenated monoterpenes being very minor. The tricyclic oxygenated sesquiterpenes (25%) were the major percentage of the total sesquiterpenes; the other sesquiterpenes included oxygenated bicyclic sesquiterpenes (about 9%), bicyclic sesquiterpene hydrocarbons (about 7%), monocyclic sesquiterpene hydrocarbons (about 6%) and tricyclic sesquiterpene hydrocarbons (about 3%). The chemical classes were not very different between the Clevenger and semi-commercial extracted *J. excelsa* EO (Table 6).

#### 2.4.2. *Juniperus sabina*—Two Different Extraction Methods (the Second Experiment)

The *J. sabina* EO profiles extracted via the Clevenger apparatus (hydrodistillation) and semi-commercial (steam distillation) were also very similar, thus refuting our hypothesis (Table 7). Fourty-five (45) EO constituents were identified in *J. sabina* from this second study, with the major one being sabinene (20.07–25.6%). The other EO constituents at > 1% of the total oil included α-thujene (1.26–1.41%), α-pinene (2.72–5.88%), β-pinene (1.92–2.73%), limonene (1.01–1.82%), γ-terpinene (2.32–2.92%), terpinen-4-ol (3.83–6.39%), myrtenyl acetate (1.36–14.20%), methyl eugenol (1.95–5.89%), γ-cadinene (1.15–2.17%), δ-cadinene (2.35–9.31%), elemol (1.32–4.53%) and germacrene-D-4-ol (1.26–5.02%) (Table 7). The main EO chemical class in both distillation methods was that of monoterpenes (Table 7). The EO from the female plants had a higher concentration of monoterpenes compared to that of the male ones. Monoterpene hydrocarbones were the main subclass of the monoterpenes (Table 7).

### 2.5. Biological Activity of the J. excelsa and J. sabina Essential Oils (EOs)

#### 2.5.1. Antimicrobial Activity

The EO of the two juniper species was tested for antimicrobial activity against 6 microorganisms using the disc diffusion method, as described in the Materials and Methods section. Overall, the EO of *J. sabina* was more effective against HI CCM4457 than that of *J. excelsa* (Table 8). However, the antimicrobial activity of the EO of the two juniper species did not differ significantly against the other five microorganisms (Table 8). In general, the best antimicrobial activity of both EOs was found against *Escherichia coli* and *Yersinia enterocolitica* (4.80 mm).

#### 2.5.2. Repellent and Insecticidal Action of the Semi-Commercial Extraction EO of *J. sabina* and *J. excelsa* on Aphids (*Sitobion avenae*, *Rhopalosiphum padi*)

The analysis of variance results on the repellent and insecticidal effects of EO from juniper species *J. sabina* (M, F) and *J. excelsa* are shown in Table 9 and Table 10. The concentration of the EO (1, 1.5, 2.5, 3.5, 4.5, and 5%) revealed a significant effect of the concentration on both aphid species (*Sitobion avenae* and *Rhopalosiphum padi*). However, the main effect of the juniper’s species and the junipers species by concentration interaction effect were not significant at the 5% level. This suggests that the repellent effect of EO concentration was consistent in *J. sabina* and *J. excelsa*, and that there was no difference between the two juniper species with respect to their repellency action. The mean mumber of aphids that remained on the leaves are shown in Table 9. The EO applied at 5% concentration had the strongest repellent action on both species of aphid. However, the 4.5% concentration rate had a stronger repellant effect on the *S. avenae* aphid than on the *Rh. padi* aphid. The two low (1% and 1.5%) concentraions did not have significantly different repellency on both species of aphid (Table 9). Overall, the number of aphids repelled increased with the increasing concentration. Surely, as expected, the EO caused visible injuries on the leaves, such as scolding, as most EO are phytotoxic. Furthermore, further research at various concentrations may be needed to establish the exact phytotoxic effect of *J. sabina* and *J. excelsa* EO on barley leaves.

#### 2.5.3. Antioxidant Capacity (ORAC) of the *J. sabina* and *J. excelsa* Essential Oils (EOs)

The antioxidant capacity of the EO from the two juniper species obtained by the two different modes of extraction (semi-commercial steam distillation and hydrodistillation via Clevenger apparatus) is shown in Table 11. The interaction effect of extraction method and species/galbuli/sex on ORAC was highly significant. Overall, when the semi-commercial steam distillation method was used, the EO from *J. excelsa* with galbuli plants had a higher antioxidant capacity (Table 11), although this result is difficult to explain precisely. The highest antioxidant capacity was exhibited by the EO from *J. excelsa* without galbuli and *J. sabina* (F) plants, both obtained via Clevenger hydrodistillation. The difference in the antioxidant capacity of the EO may be due to their differential composition. We may only speculate here, as further research may need to be conducted to reveal the precise causes.

The lowest antioxidant capacity was found in the EO of *J. sabina* (M) plants in both Clevenger hydrodistillation and SCom (semi-commercial) steam distillation (Table 11).

## 3. Discussion

### 3.1. J. excelsa and J. sabina Essential Oil (EO) Composition in Different Locations in Bulgaria and Slovakia (the First Experiment)

**Figure 1:**
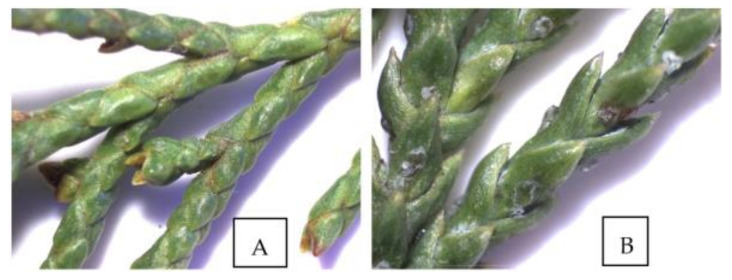
Images of the leaves of *J. sabina* (**A**) and *J. excelsa* (**B**) taken with a Stereo Microscope DM-143-FBGG, Motic Images Plus 3.0.

Botanically, *Juniperus excelsa* and *Juniperus sabina* belong to section Sabina [25], and differ from other juniper species in their scaly leaves (Figure 1). The variation of the qualitative and quantitative composition of EO of *J. excelsa* and *J. sabina* was compared using samples from populations in Bulgaria and one sample of *J. sabina* from Slovakia.

#### 3.1.1. *Juniperus excelsa*

In this study, the EO yield of the air-dried biomass varied from 0.69 to 1.87%. The highest EO of *J. excelsa* was obtained from the samples collected in the reserve “Izgoryaloto Gyune” in the Rhodope Mountains. The EO yields of the samples from Bachkovo, Rhodope Mountains and the reserve “Tisata” in the Pirin Mountains were similar. The habitats of *J. excelsa* in the reserve “Tisata” in the Pirin Mountains are under the influence of a Mediterranean climate, and in the other two locations (the reserve “Izgoryaloto Gyune” on Rodope mountain; Bachkovo, Rhodope Mountains) are under the influence of a temperate–continental climate [64]. The common denominator between the three habitats is that the species is spread on steep eroded slopes with southern and southeastern exposure, on limestone rocks. The soil layer at these locations is shallow. Previous research has demonstrated that the EO yield and composition may be influenced by the sampling time/season, the various ecological characteristics of the habitat, the sex of the plant and other factors [27,28,29,30]. The divergent results of the samples collected from populations with temperate–continental climates (the reserve “Izgoryaloto Gyune” in the Rhodope Mountains, and Bachkovo in the Rhodope Mountains), as well as the similar values of the samples under the influence of a Mediterranean climate (the reserve “Tisata” in the Pirin Mountains) suggest that the individual genetic characteristics of plants most probably predetermined the EO yield, rather than the differences in the ecology. Indeed, a significant diversity of EO yield and composition within one population was recently reported for *J. oxycedrus* L. in Bulgaria [31].

This is the first comparative study on *J. excelsa* populations in Bulgaria. Overall, the EO composition of the samples from different locations was similar, although some plants did have galbuli and others did not (please note that we removed the galbuli prior to the distillation of the EO in the first experiment). In general, the class of monoterpenes of the *J. excelsa* EO samples consisted mostly of monoterpene hydrocarbons, confirming the reports on *J. excelsa* from other studies [28,32,33,34,35,36,37,38,39,40,41,42,43,44,45,46,47,48,49,50,51,52,53,54,55,56] (Table 1). In this study, the three major EO constituents of *J. excelsa* EO were α-cedrol (29.79–32.33%), α-limonene (24.14–26.36%) and α-pinene (19.71–22.53%) (Table 3) under both the temperate–continental climate and the influence of the Mediterranean climate. This is in contradiction with the working hypothesis. A review of the previous reports on the chemical composition of *J. excelsa* EO showed significant differences in its EO composition (Table 1). For example, α-pinene was predominant in samples from Tbilisi, Georgia (40%) [33], Iran (68% [34,48,49] and 66.4%) [35], Pakistan (64%) [44], Lebanon (86.8%) [55], Turkey (29.7–34.0%, 46.1%) [45,47]; limonene (23%) and α-terpinene (24%) in samples from Oman [36,50]; cedrol (37%) in samples collected between Greece and Albania [37]; and β-terpinyl acetate (38.0%) in samples from Turkey [56] (Table 1). In a study on two populations in the Republic of North Macedonia, Sela et al. [32] reported that there were two chemotypes of *J. excelsa* EO: the pinene- and sabinene-chemotype. However, in this study, we did not identify the chemotypes of *J. excelsa*.

#### 3.1.2. *Juniperus sabina*

In this study, two populations of *J. sabina* from Bulgaria (M, F) and one population from Slovakia (M), Zvolen, were studied. According to the Red Book of Bulgaria [65], the species is categorized as critically endangered [CR] and its populations are included in the European ecological network NATURA 2000. Generally, the *J. sabina* populations in Bulgaria have a mosaic structure, a small number, low projective coverage, and poor reproducibility [66]. Plant habitats with the participation of *J. sabina* are relicts, and are located on steep slopes and on limestone and silicates rocks in the Rila Mountains, Western Stara Planina and Eastern Rhodopes, at high altitudes [39]. In this study, around 45 components were identified in *J. sabina* EO, and no significant differences were found between the samples from the tested populations. A class of monoterpenes, in particular monoterpene hydrocarbons and oxygenated monoterpenes, predominated in all of the samples. Similar results have been reported by other authors [38,39,40,41,42,43,44,45,46,47,48,49,50,51,52,53,54,55,56,57,58,59,60,61,62,63] (Table 1).

In the present study, sabinene was the principal EO constituent in male plants (24.45–30.98%) and it was in a lower concentration in the female plants (16.68%). The concentration of sabinene in the published reports varied widely from 36.8% [38], 48.6% [57] and 50.31% [60] to 70% [62].

Terpinen-4-ol, (S)-(-)-citronellic acid, methyl ester, elemol and α-sadinol were also the main EO constituents in the samples in this study, and their amounts cannot be related to the type of habitat or the sex of the plant. It is noteworthy that myrtenyl acetate was found in higher concentration in a M sample from Slovakia (20.81%) and in one sample of an F plant from Beli Iskar, Bulgaria (23.02%); its concentration in the other samples was 1.32–2.78%. In general, this study found no large differences in *J. sabina’s* EO content and composition depending on its habitat, and this refuted part of our working hypothesis.

#### 3.1.3. Podophyllotoxin

Podophyllotoxin was first reported in *J. sabina* in 1953 [67]. Hartwell et al. [67] reported a podophyllotoxin yield of 0.17% in a *J. sabina* var. *tamsciscifolia* male plant and 0.2% in the dried needles of ”Savin”, “stated by the supplier to be *J. sabina*”, while in this study, the podophyllotoxin concentration ranged from 0.065% in accession # 77 to 0.320% in accession # 65 (Table 5). These results are promising because the 0.32% podophyllotoxin in this study was the highest reported for podophyllotoxin in *J. sabina.* Therefore, accession # 77 could potentially be used for the commercial production of podophyllotoxin. Interestingly, while several papers reported on the use of podophyllotoxin’s effects and mentioned that it can be obtained from *J. sabina*, we were not able to identify any report where the podophyllotoxin yield of *J. sabina* was actually reported since 1953 (for almost 68 years). This underlines the need for more research on the podophyllotoxin yield in *J. sabina* accessions across the world.

### 3.2. Two Different Extraction Methods of EO in Juniperus excelsa and Juniperus sabina

The EO in this study was extracted from *J. excelsa* and *J. sabina* using two extraction methods: hydrodistillation (HD) methods using a Clevenger apparatus (ClevA) and steam distillation in semi-commercial extractor (SCom). The hydrodistillation (HD) method using a ClevA is the standard method for EO extraction from plant material, as described in the British Pharmacopeia [68]. Steam distillation is the predominant method for the extraction of the EO of most aromatic plants utilized in the industry because it is economical, simple and environmentally sustainable [69,70].

It is common knowledge that the composition, biological activity, aroma, color and the production cost of EOs may depend on various factors, including the type of extraction [71]. While most literature reports found significant differences in the EO composition between the hydro- and steam distillation of various plant species, and between small laboratory glassware apparatus and commercial extractors, the EO composition of *J. sabina* and *J. excelsa* in this study was very similar and not significantly different, refuting part of our working hypothesis related to the anticipated effect of different distillation methods. The concentration of the monoterpenes and sesquiterpenes in the EO was also not different between the EO obtained via the two distillation methods. The results support the notion that the extraction of small samples of *J. sabina* and *J. excelsa* via a Clevenger hydrodistillation unit will provide a representative EO content and composition of the respective oils if they are extracted via steam distillation in a commercial facility. Some previous studies reported similar findings for other aromatic plants, such as basil species [23,72], and tobacco [73].

### 3.3. Biological Activity of EO J. excelsa and J. sabina

#### 3.3.1. Antimicrobial Activity

ЕО have shown significant biological activities, such as antibacterial, antimicrobial, antifungal, antiviral and insecticidal properties, and play a notable role in allelopathic communication between plants [49,50,51]. The most sensitive bacteria to the antimicrobial activity of juniper berries EO was *Haemophilus influenzae* [32]. The findings of the latter authors were similar to our results achieved using the disc diffusion method for *H. influenza,* for which *J. sabina* EO was the most effective. The best antimicrobial activity of *J. sabina* was found against *Bacillus subtillis* and *Stapyholococcus aureus* [57]. The *J. excelsa* EO showed strong antimicrobial effects against the anaerobic bacterium *Clostridium perfringens* and moderate activity against *Staphylococcus aureus*, *Streptococcus pyogenes, Streptococcus pneumoniae, Mycobacterium smegmatis, Candida albicans* and *Candida krusei* [28]. Previously, it was reported that the *J. excelsa* EOs were active against the Gram-positive bacterium *Staphylococcus aureus* and the Gram-negative bacterium *Escherichia coli* [39].

#### 3.3.2. Repellent and Insecticidal Action of the Semi-Commercial Extraction EO of *J. sabina* and *J. excelsa* on Aphids (*Sitobion avenae, Rhopalosiphum padi*)

The aphids *Rh. padi* and *S. avenae* are economically important pests on cereals [74]. These aphids cause mechanical damage to the plants and transmit viruses, and therefore may significantly reduce the yields or compromise the entire crop [75]. The biological method for pest and disease control in agriculture is an alternative to the widely used conventional chemical production methods. EO are often used as ingredients in biopesticides because they are volatile, and therefore they are good fumigants, evaporate relatively quickly, degrade quickly in soil, and are generally less or non-toxic to animals, humans and the environment [76]. In this study, the insecticidal activity of EOs obtained from *J. sabina* (M, F) and *J. excelsa* were established by testing the EOs’ efficacy at different concentrations. The tested EOs demonstrated a very good insecticidal effect 24 h after application on the aphids (Table 10). This is due to the fact that EOs most often act as neurotoxins on the insects and they affect their physiological processes [76,77]. The EOs’ efficacy was at 100% on both aphids (*S. avenae* and *Rh. padi*). These results are not accidental, because α-рinene, sabinene, limonene and β-myrcene are the main constituents of monoterpenes in *Juniperus* species, and they have been reported to have insecticidal activity [78,79,80]. Тhe monoterpenes (α-pinene, terpineol, 1,8-cineole, limonene, α-terpinene) and phenyl propanoids (thymol and carvacrol) were found to have a high fumigant activity on *Musca domestica, Tribolium confusum* and *Sitophilus oryzae* [78,79]. These compounds of monoterpenes were also established in the juniper EOs from this study. Therefore, the results suggest that the tested EOs could be utilized at their lowest dose, 1%, to achieve a very good insecticidal effect. The results from this study suggest that the EOs of *J. sabina* (M, F) and *J. excelsa* have the potential to be used in the development of new products for the control of agricultural pests and diseases.

#### 3.3.3. Antioxidant Activity

The genus *Juniperus* comprises around 68 species and 36 different varieties which contain EO, phenols and podophyllotoxin among other non-volatile compounds [1,4]. The essential oil (EO) of juniper species has wide applications in various products. The antioxidant capacity of *J. sabina* (M, F) and *J. excelsa* EO obtained following the two extraction methods was also evaluated in this study (Table 11). The antioxidant activity of EOs is another biological property of great interest; it depends on a number of factors, such as the composition, distillation timeframe, extraction methods and plant species, among others. The antioxidant capacity of the EOs from other species of junipers (leaves and galbuli) has been previously reported [81,82,83,84]. Because the authors used different extraction methods, durations of distillations and calibrations for the comparison of the antioxidant activity, the results may not be appropriate. In this study, the highest antioxidant activity was obtained from *J. excelsa* without galbuli and *J. sabina* F by the Clevenger extraction method for 3 h distillations (Table 11). Previous research reported the antioxidant activity of *J. excelsa* galbuli [83,85] and *J. sabina* galbuli [59,83]; however, the EO in the latter studies was obtained after 4 h steam distillation, or at specific timeframes during the 4 h hydrodistillation process. Generally, EOs scavenge free radicals and may be included in products for disease protection and health maintenance [86]; however, the composition of EOs is very variable. The chemical compositions and related total antioxidant capacities of *J. excelsa* and *J. sabina* depended on many factors, such as the extraction method and the plant part, etc. Therefore, the breeding and selection of two juniper species to develop new varieties for commercial EO production would be an important step to decrease the variablility in natural sources and provide consistency in supply and quality.

## 4. Materials and Methods

### 4.1. Collection of the Plant Material for the First Experiment

**Figure 2:**
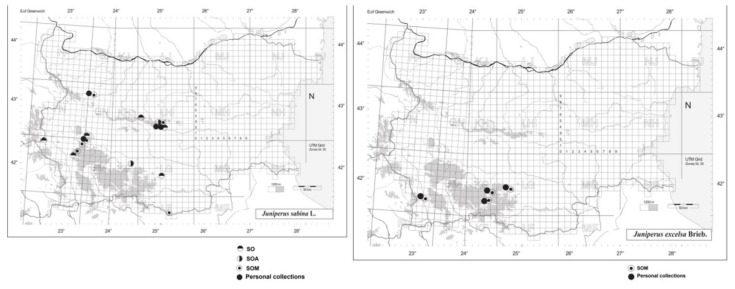
UTM map of the collection sites of *J. sabina* and *J. excelsa* in Bulgaria. SO; SOA; SOM—herbarium acronyms.

In this study, the plant materials of *Juniperus sabina* L. and *Juniperus excelsa* M. Bieb. were used. The samples of the two species were collected from populations in Bulgaria and Slovakia, as follows:

(a) *Juniperus sabina* (M, F): Krushovska bara in Stara planina (The Balkan Mountains) near the town of Vratsa, Bulgaria (43°9′55.95″ N, 23°35′16.22″ E, 678 m.a.s.l.); Beli Iskar in the Rila Mountains, Bulgaria (42°15′46″ N, 23°32′25″ E, 1,160 m.a.s.l.) (Figure 2); and Zvolen, Slovakia M from Dr. Jankovič’s garden settlement (48°34′35″ N, 19°11′23″ E; 290 to 396 m.a.s.l.).
(b) *Juniperus excelsa*: The reserve “Izgoryaloto Gyune” in the Rhodope Mountains, above the town of Krichim, Bulgaria (42°01′40″ N, 24°28′09″ E, 367 m.a.s.l.); the reserve “Tisata” in the Pirin Mountains, near Kresna town, Bulgaria (41°74′14″ N, 23°15′54″ E, 288 m.a.s.l.); and above the village of Bachkovo in the Rhodope Mountains (41°58′16″ N, 24°52′11″ E, 543 m.a.s.l.) (Figure 2).

A sampling permit was obtained from the Bulgarian Ministry of the Environment (№ 736/12 March 2018, issued to Dr. Tzenka Radoukova and Dr. Valtcho D. Zheljazkov). Voucher specimens of these species were deposited at the Herbarium of the Agricultural University, Plovdiv, Bulgaria (SOA) [87].

The leaves of the juniper samples were carefully separated, and the EO was extracted from dried samples. 

Subsamples of *J. sabina* and *J. excelsa* were also collected for podophyllotoxin analyses.

### 4.2. Essential Oil (EO) Extraction of the Juniper Biomass Samples

Two separate studies were conducted and described in this manuscript: (1) the objective of the first study was to assess the variability in the EO profile and podophyllotoxin concentration of *Juniperus sabina* and *J. excelsa,* depending on the location and sex of the tree; (2) the objective of the second experiment was to assess the effect of the EO extraction (hydrodistillation using a Clevenger-type apparatus vs. steam distillation in a semi-commercial extractor) on the EO profile and bioactivity of *J. sabina* and *J. excelsa*.

#### 4.2.1. The Hydrodistillation Extraction of the EO for the First Experiment

**Figure 3:**
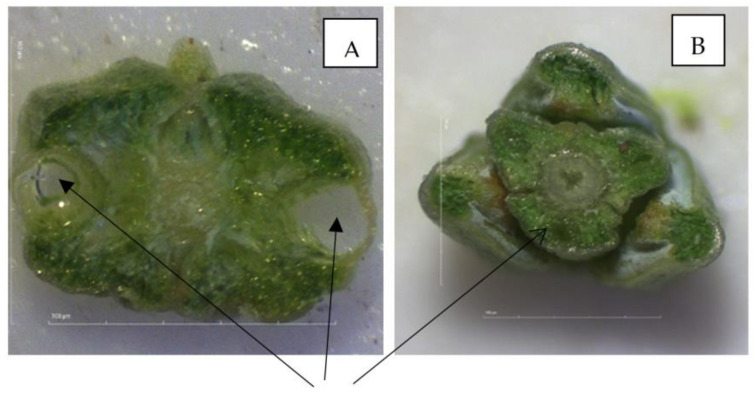
Images of the endogenic cavities of the leaves of *J. sabina* (**A**) and *J. excelsa* (**B**) taken using a Stereo Microscope DM-143-FBGG, Motic Images Plus 3.0.

All of the juniper biomass samples were dried in a shady area at a temperature below 38 °C to avoid EO losses. The EO was extracted by hydrodistillation at the University of Food Technologies in Plovdiv, Bulgaria, following a procedure described previously [83]. The subsamples for the distillation consisted of 100 g air-dried leaves, which were cut in 5 mm pieces immediately prior to the distillation. The ratio of the biomass to water was 1:10, so we used 1000 mL water for each sample. The cutting of the leaves was performed in order to facilitate the EO extraction, as juniper EO is synthesized and stored in endogenic cavities (Figure 3). The cutting was based on preliminary studies and previous reports indicating that if the juniper leaves are distilled without being cut, the distillation process may need to continue for over 10–12 h to extract all of the the EO [88,89]. The biomass material’s moisture content was determined just before the distillation by drying a subsample from each batch to a constant weight at 105 °C [68].

The EOs were extracted by hydrodistilldtion for 3 h 30 min in two replicates in a Clevenger-type laboratory glass apparatus of the British Pharmacopoeia [68], modified by Balinova and Diakov [90]. The EO obtained was dried over anhydrous sulfate and stored in tightly closed dark vials at 4 °C until these could be analyzed for their chemical profile.

#### 4.2.2. The Hydrodistillation Extraction of EO of the Second Experiment (ClevA) Distillation

The second experiment also used air-dried biomass samples of *J. excelsa* (collected at IG Krichim in the Rhodope Mountains) and *J. sabina* (collected above the village of Beli Iskar in the Rila Mountains). The EO from the second experiment was extracted at the Research Institute for Roses, Essential Oils and Medicinal Plants in the town of Kazanluk, Bulgaria through hydrodistillation. The hydrodistillation was performed in a Clevenger apparatus using 100 g air-dried samples and 800 mL water. The biomass samples were mixed in a blender in order to disrupt the EO cavities and the samples were extracted for 3 h in two replicates.

#### 4.2.3. Steam Distillation of the Samples from the Second Experiment Conducted in a Semi-Commercial Extraction Unit (SCom)

The SCom steam distillation was conducted in semi-commercial steam distillation units using leaves and small twigs, steam distilled for 3 h. The air-dried biomass sample sizes for the SCom were as follows: (1) *J. sabina*, M—4 kg; (2) *J. sabina,* F—5 kg; and (3) *J. excelsa*—5 kg.

The resulting EO from the above extractions was collected, separated from the remaining water, and kept in a freezer until the gas chromatography (GC) and mass spectroscopy (MS) analyses could be conducted. The EO was measured both by volume and by weight.

### 4.3. Quantitative Analysis of the Podophyllotoxin

The podophyllotoxin analysis was essentially performed as described previously [4], with a few modifications. The HPLC analysis was performed using an Agilent 1260 series system and an Agilent Eclipse XDB-C18, 4.6 mm × 150 mm, 5 µm column. The injection volume for all of the samples and for the podophyllotoxin standard was 10 µL. The analytical method was isocratic (28:72% acetonitrile:deionized water with 0.1% TFA) for 20 min. The analytes were detected at 220 nm. The podophyllotoxin was purchased from Sigma-Aldrich (St. Louis, MO, USA). The response factors were calculated using the equation RF = DR/C, where DR was the detector response in the peak area (PA) and C was the podophyllotoxin concentration. Confirmed integrated peaks were used to determine the percentage of podophyllotoxin in the extract. The RF of the target chemical constituent was used to determine the “percent” for each sample using the equation PA/RF/C × 100 = % (peak area/response factor/concentration) in the plant tissue (Table 5).

### 4.4. Gas Chromatography (GC) and Mass Spectrometry (MS) Analyses of the Essential Oils (EO)

The *J. excelsa* and *J. sabina* EOs in three replications were analyzed for their chemical composition by GC-FID and GC/MS, as described previously [83]. Briefly, the GC/MS analysis was performed using a 7890A gas chromatograph (Agilent Technologies Inc., Santa Clara, CA, USA) and a 5975C mass selective detector equipped with a DB-5MS capillary column with dimensions of 30 m length, 0.32 mm inner diameter and 0.25 µm film tickness (JW, Agilent) at the following temperature program: initially 60 °C for 3 min, then 1 °C/min to 80 °C (held) for 3 min, and finally 5 °C/min 280 °C (held for 5 min). The flow rate of the helium (carrier gas) was set at 1.0 mL/min. MS parameters: the ionization voltage was 70 eV; the temperatures of the ion source and transfer line were 230 and 280 °C, respectively; solvent delay 4.25 min, mass range: 50–550 Da, scan mode. In total, 1 μL EO (10%, *v*/*v* in n-hexane) was injected into the GC/MS system using split mode 25:1.

The GC analysis of the EO from the two juniper species was performed using an Agilent 7890A GC system using the same column and conditions described above in order to obtain the same elution order. The FID temperature was 270 °C.

The components present in the EO samples were identified using the mass spectra library NIST’08, and were compared with the literature data [91]. A mixture of aliphatic hydrocarbons (C_8_-C_40_) was used to calculate the relative retention indices, under the same conditions mentioned above. The normalization method of the GC/FID peak areas was used to determine the percentage ratio of the EO components.

### 4.5. Method for the Testing of the Antimicrobial Activity

#### 4.5.1. Microorganisms

The microorganisms used in this study included *Escherichia coli* CCM 3988 (EC), *Haemophilus influenzae* CCM 4457 (HI), *Shigella sonnei* CCM 1373, *Yersinia enterocolitica* CCM 5671, *Staphylococcus aureus* subs. *aureus* CCM 4223 (SS) and *Streptococcus pneumoniae* CCM 4501 (SP). All of the bacterial strains were purchased from Czech Collection of Microorganisms (Brno, Czech Republic) and were for antibacterial activity. The pure bacterial cultures were then incubated in Mueller Hinton broth (MHB, Oxoid, Basingstoke, UK) at 37 °C for 24 h.

#### 4.5.2. Disc Diffusion Method

In this study, 100 µL of the bacterial suspension was spread on the Mueller Hinton Agar (MHA, Oxoid, UK). In this study, the agar disc diffusion method was used. In total, 6 mm diameter filter paper discs were used for the test. The filter paper was impregnated with 15 μL EO and placed on MHA with a bacterial inoculum. The MHA was maintained at 4 °C for 2 h and then at 37 °C for 24 h. After a 24 h incubation period, the diameter of the inhibition zones (in mm) was measured. The antibiotic chloramphenicol (30 μg per disc, Oxoid, UK) was used as a positive control for the bacterial growth. The antimicrobial activity was measured in triplicate.

### 4.6. Methodology for the Antioxidant Capacity Evaluation of the Essential Oils (EO)

The antioxidant capacity of the different juniper EOs was measured according to the oxygen radical absorbance capacity (ORAC oil) at the University of Nebraska-Lincoln, Small Molecule Analysis Laboratory, using the method developed by Huang et al. [92,93]. Trolox®, (6-hydroxy-2,5,7,8-tetramethylchroman-2-carboxylic acid), a polar derivative of Vitamin E, was used as a standard. The EO samples were prepared by mixing 10 ± 1 mg oil with 1 mL water and acetone (1:1) with 7% methyl-β-cyclodextrins (*w*/*v*). The test of the antioxidant activity was started in a 96-well plate by first transferring 25 μL 74 mM phosphate buffer, with pH 7.4, to each well. After that, the EO sample (25 μL) or Trolox® (25 μL) was added at concentration of 0.2, 0.4, 3.3, 6.5, 10, 13, 25 or 50 μg/mL, followed by 150 μL of fluorescein (8.16 × 10^−5^ mM). Each sample was incubated at 37 °C for 10 min, with 3 min alternating shaking. The reaction was activated by adding 153 mM 2, 2′-azobis (2-amidinopropane) hydrochloride (25 μL) to each well. The standards and tested EO were prepared in 96-well plates and monitored with a BMG Labtech FLUOstar Optima microplate reader (Durham, NC). We measured the fluorescence every 1.5 min at excitation and emission wavelengths of 485 nm and 520 nm, respectively, until the decreasing fluorescence values plateaued. The area under the curve was calculated. The results are reported as µmole Trolox® g^−1^.

### 4.7. The Activity of the Semi-Commercial Extraction (Scom) EO of J. excelsa and J. sabina on the Aphids Rhopalosiphum padi (Bird Cherry—Oat Aphid) and Sitobion avenae (English Grain Aphid)

#### 4.7.1. Colonization of *Rhopalosiphum padi* and *Sitobion avenae*

The aphids *Rhopalosiphum padi* and *Sitobion avenae* were collected from cereal crops in the area of the town of Karnobat, Bulgaria (42°38′54.51″ N, 27°21′60.56″ E). The insects were reared on *Hordeum vulgáre* Jess. subsp. *distichum* L., var. Erectum, cv Obzor in pots with a diameter of 20 cm and a height of 25 cm. The aphids were colonized on the plants when the plants reached the third leaf stage. The aphid colony was maintained at a controlled temperature of 23–24 °C, 65% relative humidity and an 8:16 h (light: dark) period in the entomology laboratory of the Institute of Agriculture in Karnobat, Bulgaria. The wingless female aphids were used in the experiment.

#### 4.7.2. Repellency Tests

The repellent effect of EOs of *J. sabina* (F, M) and *J. excelsa* against *Rh. padi* and *S. avenae* were assessed using assays in Petri dishes. The EOs were used in six concentrations: 1, 1.5, 2.5, 3.5, 4.5 and 5%, in three replicates. The EOs were diluted an aqueous solution with an emulsifier, 0.1% Polysorbate 80. In total, 2 µL of each solution (1, 1.5, 2.5, 3.5, 4.5 and 5%) was applied directly to a 5 cm length of the leaves of *H. vulgare* Jess. subsp. *distichum* L., var. Erectum, cv Obzor. The leaves were dried at room temperature (about 25 °C) for 10 min and placed in Petri dishes on a wet laboratory filter paper disk. One treated leaf, one untreated leaf, plus 10 wingless aphids were introduced into each Petri dish. Then, the Petri dishes were covered with cheesecloth (44 g/m^2^). The repellent effect was observed after 24 h. The repellency of the tested EO was expressed as the number of aphids that ascended at and remained on the treated leaves.

#### 4.7.3. Testing the Insecticidal Action of the Essential Oils (EO)

The toxicity of three EO was tested on two aphid species: *S. avenae* and *Rh. padi*. The contact effect of the EO at 0, 1, 1.5, 2.5, 3.5, 4.5 and 5% concentration solution against pests was evaluated on leaves of *H. vulgare* subsp. *distichum* var. Erectum, cv Obzor, in three replicates. The EOs were diluted in an aqueous solution with an emulsifier, 0.1% Polysorbate 80. The control (0%) was treated with 0.1% aqueous solution of Polysorbate 80. The leaves of *H. vulgare* which developed colonies of aphids were cut and 2 µL of each solution was applied directly over the adult wingless forms of the aphids. After the treatment, the leaves were dried on filter paper and transferred to Petri dishes [94]. The Petri dishes were covered with cheesecloth (44 g/m^2^). The effect of the application (knock-down or mortality) was observed after 24 h and after 72 h, and the treatment effects were compared with the controls. The effect of the EO application at different concentrations was calculated using the Henderson and Tilton formula:

% Efficacy = (1 − (number of live insects in the control before treatment * number of live insects in the post-treatment variant)/(number of live insects in the pre-treatment variant * number of live insects in the pre-treatment variant)))100.

### 4.8. Statistical Analyses of the Data

The effects of the species and accession on the EO yield (results shown in Table 2) were determined using ANOVA of a nested design in which the effects in the model are the species and accession nested in species.

For *J. sabina,* the effect of accession on % (*w*/*w*) or podophyllotoxin (results shown in Table 5) was determined using one-way ANOVA.

The significance of the main and interaction effects of the extraction method and species/galbuli/sex on ORAC (results shown in Table 11) was determined using a 2 × 4 factorial design ANOVA.

In all of these ANOVA, the validity of the normal distribution and constant variance assumptions on the error terms was verified by examining the residuals. For significant effects, multiple means comparison was performed using Fisher’s LSD at a 5% level of significance, and letter groupings were generated.

## 5. Conclusions

Overall, the EO in dried leaves of *J. sabina* (1.98%) was higher than that of *J. excelsa* (1.16%). The EO content in *J. excelsa* varied from 0.69 to 1.87%, whereas the EO content in *J. sabina* was 1.3–2.1%. The main EO constituents of *J. excelsa* were α-cedrol (29.1–32.5%), α-limonene (24.1–26.4%) and α-pinene (19.7–22.5%), while those in *J. sabina* were sabinene (16.7–30.9%), terpinen-4-ol (9.3–13.6%), myrtenyl acetate (1.3–23.0%), elemol (8.5–13.7%) and α-cadinol (3.5–3.8%). The podophyllotoxin yield from the leaves of eighteen *J. sabina* accessions was 0.07–0.32% (*w*/*w*), while it was not found in *J. excelsa* accessions.

The extraction type (hydrodistillation using a Clevenger-type apparatus vs. steam distillation in a semi-commercial facility) did not significantly alter the EO composition. Overall, the EO profile of the two junipers and accessions was quite different and may be of interest to the EO industry utilizing juniper leaf essential oil. However, *J. sabina* and *J. excelsa* are protected species, and therefore their natural populations may not be utilized for the commercial production of EO of podophyllotoxin. Therefore, it is suggested that breeding and selection programs be developed with the two junipers to identify chemotypes with (1) a high EO content and desirable composition (in both junipers), and (2) a high concentration of podophyllotoxin in *J. sabina*. Such chemotypes could eventually be developed into agricultural crops that can be used as a source for the commercial production of podophyllotoxin and EO.

The statistical analyses of the repellent effects of EO from *J. sabina* (M, F) and *J. excelsa,* and the concentration of the EO (1, 1.5, 2.5, 3.5, 4.5, and 5%) revealed a significant effect of the concentration on both aphid species (*Sitobion avenae* and *Rhopalosiphum padi*). Overall, the number of aphids repelled increased with the increasing concentration of both EOs. The EO caused visible injuries on the leaves, such as scolding, as most EO are phytotoxic. Therefore, we suggest that whenever EOs are used to evaluate their activity against any organisms, the toxicity (or phytotoxicity) thresholds must be established first. The antimicrobial activity of the two juniper EOs did not differ against five microorganisms; however, overall, the strongest antimicrobial activity of both EOs was found against *Escherichia coli* and *Yersinia enterocolitica*. The EO from *J. excelsa* without galbuli and *J. sabina* (F) plants, obtained via Clevenger-type hydrodistillation, had the highest antioxidant capacity.

## Figures and Tables

**Table 1:** Summarized data of the literature on the EO of *J. excelsa* and *J. sabina*.

Author/Year	Samples	The Main EO Constituents (%)	Extr. Methods	Locality
		*Juniperus excelsa*	
Unlu et al. [28]	leaves wood	α-pinene (55.5%); α-cedrol (7.7%) sabinene (3.5%); verbenone (2.4%)	Hexane, methanol	Turkey
Shanjani et al. [30]	galbulid leaves	α-pinene (75.6–83.7%); myrcene (0.4–4.2%)	ST	Iran
Sela et al. [32]	galbuli leaves	α-pinene (33.83–70.0%); sabinene (28.52–62.0%)	ST	N. Macedonia
Chavchanidze and Kharabava [33]	leaves	α-pinene (40.2%); limonene (8.3%); sesquiterpenes (2–8%)		Tbilisi
Moein et al. [34]	leaves	α-pinene (67.71%); α-cedral (11.5%); δ-3-carene (5.19%); limonene (4.41%)	HD	Iran
Hojjati et al. [35]	leaves	α-pinene (66.4%); limonene (3.0%); myrcene (3.0%)	HD	Iran
Weli et al. [36]	galbuli	α-terpinene (23.85%); limonene (23.42%); fenchene (6.57%); camphene (6%); δ-3-carene (4.17%)	HD	Oman
Lesjak et al. [37]	leaves; galbuli	α-pinene (31–77%); cedrol (8–37%); limonene (6–15%)	HD	between Greece, Albania, Macedoni
Adams, [38]	leaves	α-pinene (26.5%); cedrol (30.8%)		Tbilisi
Khoury et al. [39]	leaves twigs	α-pinene; α-cedrol; δ-3-carene	HD	Lebanon
Zheljazkov et al. [40]	galbuli	α-pinene (52.4%); β-pinene (3.08%); β-myrcene (3.67%); limonene (7.07%); germacrene D (4.2%)	HD	Bulgaria
Thappa et al. [41]	branches leaves	sabinene limonene	ST	North America East Africa
Adams, 1990 [42,43]	leaves	cedrol (28.1%); α-pinene (22.5%) limonene (22.7%)	ST	Greece
Rafique et al. [44]	galbuli	α-pinene (64.4%); myrcene (12.4%)	alcohol	Pakistan
Topcu et al. [45]	galbuli	α-pinene (34.0%); α-cedrol (12.3%)	hexane	Turkey
Soković et al. [46]	galbuli	sabinene (72.8%)	HD	Macedonia
Topcu et al. [47]	galbuli leaves	α-pinene (29.7–34.0%); cedrol (12.3–25.3%)	HD	Turkey
Shanjani and Mirza, [48]	leaves galbuli	α-pinene (20–70%)	ST	Iran
Slehi and Mirza, [49]	leaves; galbuli	α-pinene (19.8–44%)	ST	Iran
Weli et al. [50]	leaves	α-pinene (29.7%)	HD	Oman
Almaarri et al. [51]	leaves	α-pinene (26%); germacrene B (7.63%); γ-elemene (5.66%); cedrol (3.4%)	hexane	Syria
Duran et al. [52]	galbuli	α-pinene (46.1%)		Turkey
Khajjak et al. [53]	galbuli	α-pinene; cedrol; camphene; copaene; phyllocladene; ferruginol; podocarp-7-en-3-one; pimara-8(14) 15-dien	solvent method	Balochistan
Nadir et al. [54]	leaves galbuli	β-pinene (43.4%); limonene (36.0–43.4%); sabinene (9.6–12.6%); β-phellandrene (2.7–3.9%)		Pakistan
Azzimonti et al. [55]	galbuli	α-pinene (86.8%); myrcene (3.2%)	HD	Lebanon
Yaglioglu et al. [56]	leaves galbuli	β-terpinyl acetate (38.0%); α-pinene (37.3%)	ST	Turkey
		*Juniperus sabina*	
Adams, [38]	leaves	sabinene (36.8%); cedrol (15.2%) terpinen-4-ol (4.1%)	ST	Kazakstan
Zheljazkov et al. [40]	galbuli	α-pinene (18.4%); sabinene (23.30%) β-myrcene (3.36%); α-terpinene (3.09%); limonene (4.36%); γ-terpinene (6.20%); terpinen-4-ol (11.90%); α-terpinyl acetate (3.04%)	HD	Bulgaria
Asili et al. [57]	galbuli branchlets	sabinene (24.3–48.6%; 21.5%) α-pinene (6.2–8.1%; 14.7%) myrcene (7.6–10.8%; 6.8%)	ethanol extract	Iran
Fournier et al. [58]	leaves	sabinene (18.3–40.8%); sabinyl acetate (19.1–53.1%);		France
Emami et al. [59]	galbulid leaves	sabinene (36.3–50.59%) trans-sabinyl acetate (22.07–48.2%)	ST	Iran
Esmaili et al. [60]	galbuli aerial parts	sabinene (36.59–50.31%); α-thujene (0.11–0.32%)	HD	Iran
Khani et al. [61]	aerial parts	sabinene (12.57%); α-pinene (12.02%) limonene (9.25%); myristicin (8.61%) apiol (6.28%); germacrene D (5.59%)	HD	Iran
Sampietro et al. [62]	leaves	sabinene (64%)	HD	Kazakhstan
Abdel-Kader et al. [63]	aerial parts	sabinene (55.82%); α-pinene (5.21%)	HD	Saudi Arabia

**Table 2:** Mean essential oil (EO) yield (%) obtained from the two species and the accessions nested in the species.

Species	EO Yield	Population (Species)	EO Yield
*J. excelsa*	1.16 ^b,^*	BR1 (*J. excelsa*)	1.00 ^def^
*J. sabina*	1.98 ^a^	BR2 (*J. excelsa*)	0.69 ^f^
		IG1 (*J. excelsa*)	1.87 ^bc^
		IG2 (*J. excelsa*)	1.47 ^cd^
		KG1 (*J. еxcelsa*)	0.80 ^ef^
		SKZ (*J. sabina*)	1.38 ^cde^
		BI1 (*J. sabina*)	2.73 ^a^
		BI2 (*J. sabina*)	2.28 ^ab^
		KB (*J. sabina*)	1.54 ^c^

* Within each column, means sharing the same letter are not significantly different. Abbreviations of the populations: Bachkovo, Rhodope Mountains, without galbuli—BR1; Bachkovo, Rhodope Mountains with galbuli—BR2; Izgoryaloto Gyune, Rhodope Mountains with galbuli—IG1; Izgoryaloto Gyune, Rhodope Mountains without galbuli—IG2; Kresna Gorge with galbuli—KG1; Zvolen, Slovakia, M—СКZ; Beli Iskar, M—BI1; Beli Iskar, F—BI2; Krushovska bara in Stara Planina, M—KB.

**Table 3:** Mean concentrations of α-pinene, β-pinene α-limonene, bornyl acetate, germacrene D, γ-cadinene, germacrene B, caryophyllene oxide, α-cedrol and 1-epi-cubenol obtained from Bachkovo in the Rhodope Mountains without galbuli (BR1), Bachkovo in the Rhodope Mountains with galbuli (BR2), Izgoryaloto Gyune in the Rhodope Mountains with galbuli (IG1), Izgoryaloto Gyune in the Rhodope Mountains without galbuli (IG2), and Kresna Gorge (spread out at the the Pirin and Maleshevska Mountains) with galbuli (KG1) of *Juniperus excelsa* in Bulgaria.

EO Constituent	Population and Galbuli Combination				
	BR1	BR2	IG1	IG2	KG1
α-Pinene	21.3 ^b^*	20.4 ^bc^	19.7 ^c^	22.5 ^a^	20.3 ^c^
β-Pinene	2.99 ^b^	2.86 ^c^	3.04 ^ab^	2.78 ^c^	3.13 ^a^
α-Limonene	24.1 ^c^	24.7 ^bc^	25.3 ^b^	26.15 ^a^	26.10 ^a^
Bornyl acetate	0.12 ^b^	0.12 ^c^	0.13 ^ab^	0.12 ^c^	0.13 ^a^
Germacrene D	2.28 ^b^	2.18 ^c^	2.32 ^ab^	2.12 ^c^	2.39 ^a^
γ-Cadinene	0.82 ^b^	0.78 ^c^	0.83 ^ab^	0.76 ^c^	0.86 ^a^
Germacrene B	1.38 ^b^	1.33 ^c^	1.41 ^ab^	1.29 ^c^	1.45 ^a^
Caryophyllene oxide	4.02 ^b^	3.84 ^c^	4.09 ^ab^	3.74 ^c^	4.21 ^a^
α-Cedrol	31.7 ^ab^	30.8 ^bc^	32.3 ^a^	29.1 ^d^	29.8 ^cd^
1-epi-Cubenol	2.38 ^b^	2.27 ^c^	2.42 ^ab^	2.21 ^c^	2.49 ^a^

* Within each row, means sharing the same letter are not significantly different.

**Table 4:** Mean concentration (%) of α-thujene, α-pinene, sabinene, β-myrcene, p-cymene, limonene, cis-sabinene hydrate, β-linalool, terpinen-4-ol, β-citronellol, (S)-(-)-citronellic acid methy, myrtenyl acetate, and methyl eugenol, germacrene D, δ-cadinene, α-cadinene, elemol, α-cadinol, and sclarene obtained from the female and male *Juniperus sabina* at the Beli Iskar location in Bulgaria, and male *Juniperus sabina* at the Krushovska bara location in Bulgaria.

Location Sex	Beli Iskar F	Beli Iskar M	Krushovska Bara M
α-Thujene	1.57 ^a^	1.47 ^b^	1.51 ^b^
α-Pinene	2.06 ^a^	1.94 ^b^	1.98 ^b^
Sabinene	16.7 ^c^	28.2 ^b^	31.0 ^a^
β-Myrcene	2.84 ^a^	2.66 ^b^	2.72 ^b^
p-Cymene	1.95 ^a^	1.83 ^b^	1.87 ^b^
Limonene	2.09 ^a^	1.96 ^b^	2.01 ^b^
cis-Sabinene hydrate	0.96 ^a^	0.92 ^b^	0.9 ^b^
β-Linalool	1.10 ^a^	0.23 ^b^	0.12 ^c^
Terpinen-4-ol	10.7 ^b^	12.4 ^a^	13.6 ^a^
β-Citronellol	0.94 ^a^	0.88 ^b^	0.90 ^b^
(S)-(-)-Citronellic acid, methy	4.94 ^a^	4.64 ^b^	4.74 ^b^
Myrtenyl acetate	23.02 ^a^	1.32 ^c^	2.78 ^b^
Methyl eugenol	0.07 ^b^	13.49 ^a^	0.06 ^b^
Germacrene D	0.88 ^a^	0.83 ^b^	0.85 ^b^
δ-Cadinene	1.77 ^a^	1.67 ^b^	1.70 ^b^
α-Cadinene	0.57 ^a^	0.54 ^b^	0.55 ^b^
Elemol	13.23 ^a^	8.45 ^b^	13.70 ^a^
α-Cadinol	3.77 ^a^	3.54 ^b^	3.62 ^b^
Sclarene	0.41 ^b^	1.38 ^a^	1.24 ^a^

Within each row, means sharing the same letter are not significantly different.

**Table 5:** Mean % of podophyllotoxin in the biomass (*w*/*w*) obtained from the 18 accessions of *J. sabina*. Means sharing the same letter are not significantly different.

Accession (#)	% (*w*/*w*)	Accession (#)	% (*w*/*w*)
55	0.15 ^de^ *	66	0.18 ^bc^
56	0.18 ^bc^	67	0.11 ^gh^
58	0.15 ^de^	68	0.2 ^b^
60	0.18 ^bc^	70	0.15 ^de^
61	0.17 ^bcd^	72	0.12 ^fg^
62	0.12 ^gh^	73	0.14 ^ef^
63	0.16 ^cde^	75	0.1 ^h^
64	0.17 ^cd^	76	0.11 ^gh^
65	0.32 ^a^	77	0.07 ^i^

* Within each column, means sharing the same letter are not significantly different.

**Table 6:** Average concentration *of Juniperus excelsa* constituents (% of the total oil in the dried bimoass) extracted using two different methods: hydrodistillation (in Clevenger apparatus) and steam distillation (in a semi-commercial unit).

Constituent Number	Volatile Constituents	RI	Concentration Range % (Min–Max) Clevenger Semi-Commercial	
			% of Total Oil by Total Peak Area	
1	α-Thujene	931	0.07–0.08	0.07–0.08
2	α-Pinene	939	18.90–22.20	21.07–22.30
3	Camphene	953	0.12–0.15	0.12–0.15
4	Sabinene	969	0.07–0.10	0.07–0.10
5	β-Pinene	974	0.37–0.41	0.36–0.41
6	β-Myrcene	991	0.90–1.00	0.86–1.00
7	α-Terpinene	1018	0.12–0.14	0.11–0.13
8	α-Limonene	1031	23.70–27.50	23.23–26.70
9	γ-Terpinene	1062	0.48–0.51	0.45–0.50
10	α-Terpinolene	1088	0.45–0.49	0.43–0.50
11	β-Linalool	1096	0.16–0.22	0.15–0.22
12	Terpinen-4-ol	1177	0.36–0.41	0.34–0.62
13	α-Terpineol	1189	0.09–0.11	0.09–0.11
14	Bornyl acetate	1285	0.09–0.13	0.09–0.13
15	Myrtenyl acetate	1298	0.09–0.11	0.08–0.11
16	α-Cubebene	1351	0.51–0.58	0.49–0.58
17	Methyl eugenol	1357	0.32–0.45	0.41–0.45
18	α-Copaene	1376	0.37–0.41	0.35–0.40
19	β-Elemene	1390	0.38–0.42	0.37–0.42
20	α-Cedrene	1414	1.90–2.18	1.80–2.17
21	β-Caryophyllene	1419	1.18–2.26	1.14–2.27
22	β-Cedrene	1424	0.89–1.04	0.85–1.04
23	γ-Elemene	1433	1.00–1.22	0.95–1.22
24	α-Humulene	1454	0.63–0.81	0.62–0.82
25	γ-Muurolene	1479	0.60–0.70	0.57–0.69
26	Germacrene D	1480	2.11–2.90	2.05–2.91
27	γ-Cadinene	1513	0.72–1.07	0.70–1.07
28	δ-Cadinene	1524	2.82–3.23	2.74–3.37
29	Germacrene B	1556	0.65–0.93	0.63–0.94
30	Caryophyllene oxide	1579	3.07–3.24	2.98–3.26
31	α-Cedrol	1598	24.06–25.55	25.00–27.00
32	1-epi-Cubenol	1627	1.92–2.29	1.86–2.30
33	γ-Eudesmol	1629	0.71–0.89	0.69–0.90
34	tau-Cadinol	1634	0.90–1.04	0.87–1.05
35	tau-Muurolol	1638	0.54–1.23	0.52–1.24
36	α-Eudesmol	1644	0.69–1.21	0.49–1.21
**Class**			% of Total Oil by Total Peak Area	
Monoterpene hydrocarbons			49.13	48.85
Oxygenated monoterpenes			0.67	0.76
Bicyclic oxygenated monoterpenes			0.21	0.21
**Total monoterpenes**			**50.01**	**49.82**
Monocyclic sesquiterpene hydrocarbons			5.75	5.59
Oxygenated bicyclic sesquiterpenes			8.87	8.57
Tricyclic sesquiterpene hydrocarbons			3.01	2.93
Bicyclic sesquiterpene hydrocarbons			7.01	6.87
Tricyclic oxygenated sesquiterpenes			24.80	25.90
**Total sesquiterpenes**			**49.43**	**49.86**
Phenylpropanoid compound			0.38	0.42

**Table 7:** Average concentration of the 45 *Juniperus sabina* constituents (% of the total oil in the dried bimoass) extracted using two different methods: hydrodistillation (in a Clevenger apparatus) and steam distillation (in a semi-commercial unit).

Constituent Number	Volatile Constituents	RI	Concentration Range % (Min–Max) Clevenger Commercial			
			% of Total Oil by Total Peak Area			
1	α-Thujene	931	1.28–1.41		1.26–1.41	
2	α-Pinene	939	2.78–5.87		2.72–5.88	
3	Camphene	953	0.09–0.11		0.08–0.11	
4	Sabinene	969	20.48–24.57		20.07–25.63	
5	β-Pinene	974	1.96–2.73		1.92–2.73	
6	β-Myrcene	991	0.53–0.89		0.52–0.89	
7	α-Terpinene	1018	0.89–1.83		0.87–1.831	
8	p-Cymene	1025	0.41–1.02		0.41–1.03	
9	Limonene	1031	1.03–1.82		1.01–1.82	
10	β-Ocimene	1050	0.33–0.72		0.32–0.73	
11	γ-Terpinene	1062	2.37–2.92		2.32–2.92	
12	α-Terpinolene	1088	0.77–1.00		0.76–1.06	
13	cis-Sabinol	1090	0.11–6.81		0.11–6.82	
14	α-Thujone	1098	0.22–2.08		0.22–2.08	
15	Chrysanthone	1125	0.21–2.29		0.20–2.29	
16	Terpinen-4-ol	1177	3.90–6.38		3.83–6.39	
17	β-Citronellol	1225	0.56–0.80		0.55–0.80	
18	Linalyl acetate	1257	0.19–0.21		0.18–0.21	
19	(S)-(-)-Citronellic acid. methyl ester	1262	2.28–2.89		2.23–2.90	
20	Bornyl acetate	1285	0.87–1.04		0.85–1.04	
21	Myrtenyl acetate	1298	1.39–14.10		1.36–14.20	
22	δ-Elemene	1338	0.38–0.48		0.38–0.48	
23	Methyl eugenol	1357	1.95–5.88		2.31–5.89	
24	β-Elemene	1390	0.73–0.83		0.71–0.83	
25	β-Caryophyllene	1419	0.37–0.63		0.36–0.63	
26	γ-Elemene	1433	0.27–0.39		0.26–0.39	
27	α-Humulene	1454	0.17–0.25		0.16–0.25	
28	γ-Muurolene	1479	0.21–0.32		0.21–0.32	
29	Germacrene D	1480	1.38–1.69		1.36–1.69	
30	α-Muurolene	1500	0.48–2.39		0.47–2.39	
31	γ-Cadinene	1513	1.17–2.16		1.15–2.17	
32	δ-Cadinene	1524	2.40–9.29		2.35–9.31	
33	α-Cadinene	1538	0.38–0.45		0.37–0.45	
34	Elemol	1549	1.34–4.52		1.32–4.53	
35	Germacrene-D-4-ol	1575	1.28–4.50		1.26–5.02	
36	Spathulenol	1578	0.12–0.22		0.12–0.22	
37	δ-Cadinol	1619	0.38–0.67		0.37–0.67	
38	γ-Eudesmol	1629	0.43–0.69		0.42–0.69	
39	tau-Cadinol	1634	0.38–2.85		0.37–2.85	
40	tau-Muurolol	1638	0.33–2.90		0.33–2.91	
41	α-Cadinol	1641	0.72–8.34		0.71–8.35	
42	β-Eudesmol	1651	0.28–1.06		0.27–1.06	
43	α-Eudesmol	1644	0.36–0.61		0.35–0.67	
44	Farnesol	1692	0.49–1.04		0.48–1.04	
45	Farnesal	1707	0.51–0.89		0.50–0.89	
**Class**			**Clevenger**		**Commercial**	
			**Male**	**Female**	**Male**	**Female**
Monoterpene hydrocarbons			34.60	41.80	34.30	42.40
Phenolic monoterpenes			0.41	1.02	0.41	1.01
Oxygenated monoterpenes			7.10	15.29	7.04	15.14
Phenylpropanoid compound			5.88	1.95	5.83	2.33
Ester of monoterpenoid carboxylic acid			2.89	2.28	2.87	2.26
Acyclic oxygenated monoterpenes			0.56	0.80	0.56	0.79
Bicyclic oxygenated monoterpenes			2.26	15.18	2.24	15.04
**Total monoterpenes**			**53.70**	**78.30**	**56.10**	**79.00**
Oxygenated bicyclic sesquiterpenes			17.11	2.15	16.98	2.84
Monocyclic sesquiterpene hydrocarbons			3.06	3.09	3.00	3.06
Bicyclic sesquiterpene hydrocarbons			14.97	5.69	14.83	5.65
Monocyclic oxygenated sesquiterpenes			5.80	5.84	5.75	6.29
Tricyclic oxygenated sesquiterpenes			0.22	0.12	0.22	0.12
Acyclic sesquiterpene hydrocarbons			1.93	1.00	1.91	0.99
**Total sesquiterpenes**			**42.9**	**17.8**	**42.5**	**18.9**
**Monocyclic diterpenes**			**1.22**	**0.12**	**1.21**	**0.11**

**Table 8:** Antimicrobial activity of juniperus with the disc diffusion method in mm and the microdilution broth method in µL/mL.

Antimicrobial Activity	*J. excelsa*	*J. sabina*	ATB (C)	*J. excelsa*		*J. sabina*	
	Mean ± SD	Mean ± SD		MIC50	MIC90	MIC50	MIC90
*Escherichia coli* CCM3988	4.00 ± 1.47	5.86 ± 3.31	23.33 ± 1.53	28.36 ± 0.36	34.31 ± 0.19	17.82 ± 0.15	24.55 ± 0.09
*Haemophilus influenzae* CCM4457	3.56 ± 1.46	5.24 ± 2.57	23.67 ± 0.58	34.56 ± 0.09	42.83 ± 0.45	24.57 ± 0.12	31.28 ± 0.04
*Shigella sonnei* CCM1373	4.03 ± 1.65	5.71 ± 3.65	24.67 ± 0.58	28.31 ± 0.33	34.15 ± 0.21	16.84 ± 0.04	23.29 ± 0.05
*Yersinia enterocolitica* CCM5671	3.89 ± 1.63	6.19 ± 3.90	20.33 ± 0.58	26.36 ± 0.41	32.31 ± 0.15	15.86 ± 0.35	23.56 ± 0.05
*Streptococcus pneumoniae* CCM4501	3.44 ± 1.36	6.14 ± 3.61	22.00 ± 1.00	35.64 ± 0.15	43.21 ± 0.54	15.46 ± 0.65	23.35 ± 0.67
*Staphylococcus aureus* CCM4223	3.67 ± 1.10	3.38 ± 1.40	24.67 ± 0.58	41.26 ± 0.48	52.36 ± 0.25	49.59 ± 0.28	51.33 ± 0.78

C—chloramphenicol.

**Table 9:** Mean number of *Sitobion avenae* and *Rhopalosiphum padi* species of aphid that remained on the leaves from the six (6) concentrations of the essential oil (EO) of *J. sabina* and *J. excelsa*.

Concentration (%)	*S. avenae* Remained	*R. padi* Remained
1.0	3.89 ^a^	4.11 ^a^
1.5	3.11 ^ab^	3.56 ^ab^
2.5	2.00 ^bc^	1.89 ^c^
3.5	2.00 ^bc^	2.78 ^bc^
4.5	1.44 ^cd^	3.00 ^abc^
5.0	0.33 *^d^	0.44 *^d^

* The fewer aphids per leaf, the stronger the repellent effect. Within each column, means sharing the same letter are not significantly different at the 5% level of significance.

**Table 10:** Insecticidal action of the semi-commercial extraction of the essential oils (EOs) of *J. sabina* and *J. excelsa* on two aphid species.

EO/% Concentration	nb Aphids before Treatment		After Treatment/h							
	*Sitobion avenae*	*Rhopalosiphum padi*	*Sitobion avenae*				*Rhopalosiphum padi*			
			24 h		72 h		24 h		72 h	
			nb/Alive	E%	nb/Alive	E%	nb/Alive	E%	nb/Alive	E%
*J. sabina* M										
5%	28	38	0	100	0	100	0	100	0	100
4.5%	32	27	0	100	0	100	0	100	0	100
3.5%	31	26	0	100	0	100	0	100	0	100
2.5%	28	27	0	100	0	100	0	100	0	100
1.5%	20	28	0	100	0	100	0	100	0	100
1%	33	38	0	100	0	100	0	100	0	100
*J. sabina* F										
5%	26	24	0	100	0	100	0	100	0	100
4.5%	26	26	0	100	0	100	0	100	0	100
3.5%	36	35	0	100	0	100	0	100	0	100
2.5%	21	26	0	100	0	100	0	100	0	100
1.5%	34	30	0	100	0	100	0	100	0	100
1%	28	28	0	100	0	96.5	0	100	0	100
*J. excelsa*										
5%	28	37	0	100	0	100	0	100	0	100
4.5%	27	26	0	100	0	100	0	100	0	100
3.5%	29	36	0	100	0	100	0	100	0	100
2.5%	24	33	0	100	0	100	0	100	0	100
1.5%	28	27	0	100	0	100	0	100	0	100
1%	20	25	0	100	0	92	0	100	0	100
Control	27	34	27		27		34		34	

E%—efficacy.

**Table 11:** Mean ORAC (μmol Trolox® g^−1^) obtained from the eight combinations of the extraction method and species/galbuli/sex.

Extraction Method	Species, Galbuli, Sex	ORAC
Clevenger	*J. excelsa* with galbuli	99.1 ^b^*
Clevenger	*J. excelsa* without galbuli	165.5 ^a^
Clevenger	*J. sabina* F	166.3 ^a^
Clevenger	*J. sabina* M	41.5 ^d^
SCom	*J. excelsa* with galbuli	101.3 ^b^
SCom	*J. excelsa* without galbuli	64.1 ^c^
SCom	*J. sabina* F	71.9 ^c^
SCom	*J. sabina* M	23.9 ^d^

* Means sharing the same letter are not significantly different at the 5% level.

## Data Availability

Data of the compounds are available from the authors.

## Supplementary Materials

The following are available online, Supplementary Table S1. Average concentration of volatile constituents (% in air-dried biomass) of *Juniperus excelsa* collected in Bulgaria. Supplementary Table S2. Average concentration of volatile constituents (% of total oil in dried bimoass) of *Juniperus sabina* collected in Bulgaria and in Slovakia.
