# Supplementary material for: Essential Oil Composition and Bioactivity of Two Juniper Species from Bulgaria and Slovakia
Source: Molecules. 2021 Jun 15;26(12):3659. doi: 10.3390/molecules26123659 (PMC8232667; doi:10.3390/molecules26123659)
Supplement: Supplementary file 1 [file molecules-26-03659-s001.zip › molecules-1218267-supplementary.pdf]

Essential oil composition and bioactivity of two Junipers species from Bulgaria and Slovakia

Valtcho D. Zheljazkov [1*], Charles L. Cantrell[2], Ivanka Semerdjieva[3], Tzenka Radoukova[4], Albena Stoyanova[5], Vasilina Maneva[6], Miroslava Kačaniova[7,8], Tess Astatkie[9], Daniela Borisova[10], Ivayla Dincheva[11], Ivan Salamon[12]

Supplemental Tables

Supplemental Table 1. Average concentration of volatile constituents (% in air-dried biomass) of *Juniperus excelsa* collected in Bulgaria.

Constituent number	Volatile constituents of *J. excelsa*	RI	BR1	BR2	IG1	IG2	KG1
			--- % of total oil by total peak area ------				
1	α-Thujene	931	0.07	0.07	0.07	0.07	0.07
2	α-Pinene	939	20.4	21.31	19.71	22.53	20.32
3	Camphene	953	0.40	0.42	0.43	0.39	0.44
4	Sabinene	969	0.03	0.04	0.04	0.03	0.04
5	β-Pinene	974	2.86	2.99	3.04	2.77	3.13
6	β-Myrcene	991	0.05	0.05	0.05	0.05	0.05
7	α-Terpinene	1018	0.03	0.04	0.04	0.03	0.04
8	α-Limonene	1031	24.73	24.14	25.33	26.15	26.36
9	γ-Terpinene	1062	0.23	0.25	0.25	0.23	0.26
10	α-Terpinolene	1088	0.17	0.18	0.18	0.16	0.19
11	β-Linalool	1096	0.05	0.05	0.05	0.05	0.05
12	Terpinen-4-ol	1177	0.60	0.63	0.64	0.58	0.66
13	α-Terpineol	1189	0.33	0.35	0.36	0.33	0.37
14	Bornyl acetate	1285	0.11	0.12	0.12	0.12	0.13
15	α-Cubebene	1351	0.58	0.61	0.61	0.56	0.64
16	α-Copaene	1376	0.21	0.22	0.22	0.21	0.24
17	β-Bourbunene	1384	0.10	0.10	0.10	0.09	0.11
18	β-Elemene	1390	0.21	0.22	0.22	0.20	0.23
19	β-Caryophyllene	1419	0.43	0.44	0.45	0.41	0.46
20	γ-Elemene	1433	0.35	0.37	0.37	0.34	0.38
21	α-Humulene	1454	0.22	0.22	0.23	0.21	0.24
22	γ-Muurolene	1479	0.06	0.06	0.06	0.06	0.07
23	Germacrene D	1480	2.18	2.28	2.30	2.12	2.39
24	γ-Cadinene	1513	0.77	0.81	0.83	0.76	0.85
25	δ-Cadinene	1524	3.54	3.33	3.40	3.10	3.50
26	Germacrene B	1556	1.33	1.39	1.41	1.29	1.45
27	Caryophyllene oxide	1579	3.84	4.02	3.89	3.74	4.21
28	α-Cedrol	1598	30.83	31.71	32.33	29.09	29.79
29	1-epi-Cubenol	1627	2.27	2.38	2.42	2.21	2.49
**Class**			**% of total oil by total peak area**				
Monoterpene hydrocarbons			48.80	49.48	49.16	52.68	50.66

Oxygenated monoterpenes	1.10	1.16	1.18	1.08	1.21
**Total monoterpenes**	**49.92**	**50.64**	**50.34**	**53.76**	**51.87**
Monocyclic sesquiterpene hydrocarbons	8.80	9.20	9.41	8.57	9.66
Bicyclic sesquiterpene hydrocarbons	5.03	4.89	4.98	4.55	5.13
Tricyclic oxygenatedsesquiterpenes	30.83	31.71	32.33	29.09	29.79
Oxygenatedbicyclic sesquiterpenes	2.27	2.376	2.42	2.21	2.49
**Total sesquiterpenes**	**46.95**	**48.18**	**49.14**	**44.42**	**47.08**

Abbreviations of population: Bachkovo, Rodopi, without galbuli – BR1; Bachkovo, Rodopi with galbuli – BR2; IG Krichim, Rodopi with galbuli– IG1; Izgoryaloto Gyune, Rodopi without galbuli – IG2; Kresna gorge with galbuli – KG1.

Supplemental Table 2. Average concentration of volatile constituents (% of total oil in dried bimoass) of *Juniperus sabina* collected in Bulgaria and in Slovakia.

**Constituent number**	**Volatile constituents of *J. sabina***	**RI**	**Zvolen, SK**	**BI1**	**BI2**	**KB**
			-- % of total oil by total peak area --			
1	1-Hexanal	809	0.22	0.23	0.24	0.23
2	2-Hexen-1-al	860	0.03	0.03	0.03	0.03
3	3-Hexen-1-ol	868	0.04	0.04	0.04	0.03
4	α-Thujene	931	1.45	1.47	1.57	1.51
5	α-Pinene	939	1.89	1.94	2.06	1.97
6	Camphene	953	0.07	0.07	0.08	0.07
7	Sabinene	969	24.45	28.22	16.68	30.98
8	β-Pinene	974	0.15	0.15	0.16	0.15
9	β-Myrcene	991	2.61	2.66	2.84	2.72
10	α-Terpinene	1018	0.32	0.33	0.35	0.34
11	p-Cymene	1025	1.79	1.83	1.95	1.88
12	Limonene	1031	1.93	1.96	2.09	2.00
13	β-Ocimene	1050	0.44	0.45	0.48	0.46
14	γ-Terpinene	1062	0.51	0.53	0.56	0.53
15	*cis*-Sabinene hydrate	1068	0.88	0.90	0.96	0.92
16	α-Terpinolene	1088	0.43	0.44	0.05	0.88
17	β-Linalool	1096	0.12	0.23	1.10	0.12
18	*trans* Sabinene hydrate	1098	0.64	0.65	0.70	0.67
19	*cis*-Thujone	1102	0.20	0.21	0.21	0.21
20	*trans*-Thujone	1114	0.11	0.12	0.12	0.12
21	Terpinen-4-ol	1177	9.25	12.41	10.66	13.63
22	β-Citronellol	1225	0.87	0.88	0.94	0.90
23	Linalyl acetate	1257	0.25	0.25	0.27	0.25
24	(S)-(-)- Methyl citronellate	1262	4.55	4.64	4.94	4.74
25	Bornyl acetate	1285	0.12	0.12	0.13	0.12
26	Myrtenyl acetate	1298	20.81	1.32	23.02	2.78
27	δ-Elemene	1338	0.32	0.32	0.34	0.33
28	Methyl eugenol	1357	0.06	13.49	0.06	0.06
29	β-Elemene	1390	0.29	0.29	0.31	0.30

30	β-Caryophyllene	1419	0.11	0.10	0.16	0.27
31	γ-Elemene	1433	0.49	0.50	0.53	0.51
32	α-Humulene	1454	0.20	0.21	0.22	0.21
33	γ-Muurolene	1479	0.09	0.08	0.09	0.09
34	Germacrene D	1480	0.81	0.83	0.88	0.85
35	α-Muurolene	1500	0.27	0.27	0.29	0.28
36	γ-Cadinene	1513	0.64	0.65	0.70	0.67
37	δ-Cadinene	1524	1.63	1.67	1.77	1.70
38	α-Cadinene	1538	0.53	0.54	0.57	0.55
39	Elemol	1549	12.18	8.45	13.23	13.70
40	Germacrene-D-4-ol	1575	0.13	0.13	0.56	0.13
41	Spathulenol	1578	0.51	0.52	0.55	0.53
42	γ-Eudesmol	1632	0.68	0.69	0.73	0.70
43	β-Eudesmol	1651	0.54	0.56	0.59	0.56
44	α-Cadinol	1641	3.47	3.54	3.77	3.62
45	α-Eudesmol	1654	0.61	0.62	0.66	0.63
46	Sclarene	1974	0.38	1.38	0.41	1.24
**Class**			% of total oil by total peak area			
Oxygenated aliphatic			0.29	0.29	0.31	0.30
Monoterpene hydrocarbons			34.26	38.00	26.92	41.50
Oxygenated monoterpenes			31.72	15.55	36.50	18.15
Other monoterpenes			7.23	7.70	7.21	8.20
**Total monoterpenes**			**73.20**	**61.26**	**70.63**	**67.94**
Monocyclic sesquiterpene hydrocarbons			14.22	10.52	15.88	15.81
Bicyclic sesquiterpene hydrocarbons			3.46	3.53	3.76	3.78
Oxygenated bicyclic sesquiterpenes			5.29	5.40	5.75	5.51
Other sesquiterpenes			0.51	0.52	0.55	0.52
**Total sesquiterpenes**			**23.48**	**19.97**	**25.96**	**25.63**
Phenylpropanoid compound			0.06	13.50	0.07	0.07

Abbreviations of populations: Zvolen, Slovakia, M – CK; Beli Iskar, M – BI1; Beli Iskar, F – BI2; Krushovska bara, Stara planina, M – KB.
